# Visfatin Amplifies Cardiac Inflammation and Aggravates Cardiac Injury via the NF-*κ*B p65 Signaling Pathway in LPS-Treated Mice

**DOI:** 10.1155/2022/3306559

**Published:** 2022-10-10

**Authors:** Yewen Hu, Nan Wu, Weiping Du, Shuangshuang Wang, Jian Wang, Chaoxia Zhang, Xiaomin Chen, Caijie Shen

**Affiliations:** ^1^Department of Cardiology, Ningbo First Hospital, Ningbo, 315000 Zhejiang Province, China; ^2^Key Laboratory of Precision Medicine for Atherosclerotic Diseases of Zhejiang Province, Ningbo, 315000 Zhejiang Province, China; ^3^Department of Cardiology, Wenling First People's Hospital, The Affiliated Wenling Hospital of Wenzhou Medical University, Wenling, 317500 Zhejiang Province, China

## Abstract

**Background:**

Visfatin is an adipocytokine that has been demonstrated to be involved in cardiovascular diseases. This study aims at determining the role of visfatin in sepsis-induced cardiac injury and identify its possible mechanisms.

**Methods:**

Dynamic changes in visfatin expression in mice with lipopolysaccharide- (LPS-) induced septicemia were measured. Additionally, mice were pretreated with visfatin and further administered LPS to observe the effects of visfatin on cardiac injury. Finally, septic mice were also pretreated with JSH-23 to investigate whether visfatin regulates cardiac injury via the NF-*κ*B p65 pathway.

**Results:**

Visfatin expression levels in both the heart and serum were increased in LPS-treated mice and peaked at 6 hours, and visfatin was derived from cardiac macrophages. In septic mice, pretreatment with visfatin reduced the survival rate, worsened cardiac dysfunction, and increased the expression of cardiac injury markers, including creatine kinase myocardial bound (CK-MB) and lactate dehydrogenase (LDH). Treatment with visfatin also increased the infiltration of CD3+ cells and F4/80+ cells, amplified the cardiac inflammatory response, and elevated myocardial cell apoptosis. Treatment with JSH-23 reversed the effects of visfatin in septic mice.

**Conclusions:**

This study showed that visfatin amplifies the cardiac inflammatory response and aggravates cardiac injury through the p65 signaling pathway. Visfatin may be a clinical target for preventing cardiac injury in sepsis.

## 1. Introduction

Sepsis is a systemic inflammatory syndrome caused by infection, and patients who do not receive timely treatment can progress to multiple organ failure [1, 2]. Among the complications observed in septic patients, cardiac injury and subsequent cardiac dysfunction are the most serious, and the mortality is extremely high and may reach 90% [3]. Therefore, it is beneficial to find new intervention methods to improve cardiac injury and cardiac dysfunction in the treatment of patients with sepsis.

Visfatin, which is considered a secreted adipokine and was first discovered and subsequently isolated in 2005, can be expressed in tissues and organs of many mammals [4]. Visfatin is mainly released by adipocytes, macrophages, and skeletal muscle cells through the endoplasmic reticulum or microvesicles [4, 5]. A variety of pathological conditions, including aging, oxidative stress, a high-fat diet and inflammatory responses, can promote the release of visfatin, and macrophages are the main source of visfatin in inflammatory environments [6–9].

Previous studies also showed that visfatin is closely related to cardiovascular diseases. Downregulation of visfatin expression can significantly improve endothelial dysfunction and inhibit the inflammatory response, thus reducing the aortic plaque area in ApoE-/- mice, while treatment with exogenous visfatin aggravated the inflammatory response and promoted the progression of atherosclerosis [10–12]. Circulating levels of visfatin were increased in patients with acute myocardial infarction, and elevated visfatin expression suggested a higher incidence of major adverse cardiovascular events [13]. In addition, visfatin is highly expressed in patients with refractory hypertension, and its expression level is positively correlated with the severity of left ventricular hypertrophy [14]. Nevertheless, the role of visfatin in sepsis-induced cardiac injury remains unknown. In this study, the role of visfatin in cardiac injury and the possible mechanisms were investigated in septic mice.

## 2. Methods and Materials

### 2.1. Mice and Mouse Septicemia Models

Wild-type male C57BL/6 J mice (WT, GemPharmatech, China) aged 9-10 weeks were used in this study. First, WT mice were intraperitoneally (IP) injected with lipopolysaccharide (LPS, 10 mg/kg, Sigma) or saline, and visfatin expression at different time points and in samples was measured. Second, WT mice were pretreated with PBS or visfatin (100 *μ*g/kg, Adipo Bioscience) for 30 minutes [15] and then IP injected with LPS or saline for 6 hours; the effects of cardiac injury were detected in each group. Some of the mice above were observed for 8 days, and the mortality rates were recorded. Finally, they were pretreated with PBS + JSH-23 (3 mg/kg) [16], visfatin+JSH-23, DMSO+PBS, or DMSO+visfatin for 30 minutes, and then all mice received LPS for 6 hours. Some mice were followed up for 8 days to observe the survival rate.

The mice were euthanized at the end of the treatment, and then the blood and heart were collected separately. The supernatant was obtained after the blood samples were centrifuged at 3000 × *g* for 15 minutes and stored in liquid nitrogen. The heart samples were divided into two parts and stored in 4% paraformaldehyde on liquid nitrogen. This study was approved by the Institutional Animal Care and Use Committee of our hospital (approval NO. cardiac 20200123a).

### 2.2. Detection of Cardiac Function

Both echocardiography and hemodynamics were used to evaluate the cardiac function of each mouse. After the mice were IP injected with LPS or saline for 6 hours, they were anesthetized with 1.5% isoflurane and then placed flat on the operating table. Echocardiography with a 10-MHz linear array ultrasound transducer that included a MyLab 30CV ultrasound system (Esaote SpA, Italy) was used to obtain information on the left ventricle end-diastolic diameter (LVEDD), left ventricle end-systolic diameter (LVESD), left ventricle ejection fraction (LVEF), and fractional shortening (FS). Then, a microtip catheter transducer (Millar, Inc., USA) was inserted into the left ventricle via the right carotid artery, and the maximal slope values of the systolic pressure increment (+dp/dt) and diastolic pressure decrement (−dp/dt) were recorded using a Millar PressureVolume system (Millar, Inc.).

### 2.3. Detection of mRNA Expression Levels

The left ventricular tissue was removed from liquid nitrogen, ground into a powder, and then extracted with TRIzol Reagent (Roche). After the total RNA was collected, the concentration was determined, and 2 *μ*g of total RNA was used to synthesize cDNA using a reverse transcription kit (Roche) according to the manufacturer's instructions. Then, the cDNA was used to perform PCR amplification with LightCycler 480 SYBR Green Master Mix (Roche) to detect the expression levels of target mRNAs, including visfatin, monocyte chemotactic protein-1 (MCP-1), tumor necrosis factor-*α* (TNF-*α*), interferon-*γ* (IFN-*γ*), IL-1*β*, IL-6, IL-12, IL-17, and IL-18, in each group; the expression levels of target mRNAs were measured and normalized to GAPDH mRNA levels. The primers used are listed in Table 1.

### 2.4. Analysis of NF-*κ*B p65 Pathway Phosphorylation

Left ventricular tissue was lysed using radioimmunoprecipitation assay lysis buffer, and then the supernatant was collected as total protein. The concentration of each sample was determined using a BCA Protein Assay Kit (Thermo Fisher Scientific) and then quantified to the same concentration. Then, approximately 45 *μ*g of total protein was used to perform electrophoresis on Laemmli sodium dodecyl sulfate (SDS) polyacrylamide gels to separate proteins of different molecular weights. After transfer to Immobilon-FL PVDF membranes (Millipore), the membranes were blocked with 5% nonfat milk and then incubated with anti-NF-*κ*B p65, anti-NF-*κ*B p65, anti-cleaved caspase3, anti-Bcl2, and anti-GAPDH (all purchased from Abcam) antibodies at 4°C overnight. Then, the membranes were incubated with secondary antibodies and scanned using the Odyssey (LI-COR Biosciences).

### 2.5. Measurement of Visfatin, Cardiac Injury Markers, and Inflammatory Cytokines

The serum and the total protein collected from the left ventricle were diluted; mouse visfatin enzyme-linked immunosorbent assay (ELISA) kits were used to detect circulating and cardiac visfatin levels, and creatine kinase myocardial bound (CK-MB) assay kits and lactate dehydrogenase (LDH) assay kits were used to detect CK-MB and LDH expression in both serum and supernatant. In addition, the expression levels of MCP-1, TNF-*α*, IFN-*γ*, IL-1*β*, IL-6, IL-12, IL-17, and IL-18 in the serum were also detected using ELISA kits. All experimental procedures were carried out in accordance with the manufacturer's instructions.

### 2.6. Histological Analysis

The heart tissue was fixed with 4% paraformaldehyde for two days, embedded in paraffin, cut into 4-6 *μ*m sections, and further mounted on slides. Slides were incubated with mouse anti-visfatin, mouse anti-CD3, and anti-CD68 antibodies (all three purchased from GeneTex, USA) to determine the cardiac abundance of visfatin, T lymphocytes, and macrophages. Some of the slides were incubated with both anti-visfatin and anti-F4/80 antibodies to determine whether visfatin is secreted by cardiac macrophages. Apoptosis of myocardial cells was measured using terminal deoxynucleotidyl transferase-mediated dUTP nick end labeling (TUNEL) staining kits (Millipore, USA) according to the manufacturer's instructions.

### 2.7. Data Analysis

Data in this study are expressed as the mean ± SD, and all data were analyzed using GraphPad Prism 7. Student's *t*-test was performed to analyze differences in the means of 2 groups, and one-way and two-way ANOVA was used to evaluate differences among 3 or more groups. Survival rates during the 8-day follow up were analyzed using the log-rank test. *p* < 0.05 was considered statistically significant.

## 3. Results

### 3.1. Visfatin Levels Are Increased in Septic Mice

**Figure 1:**
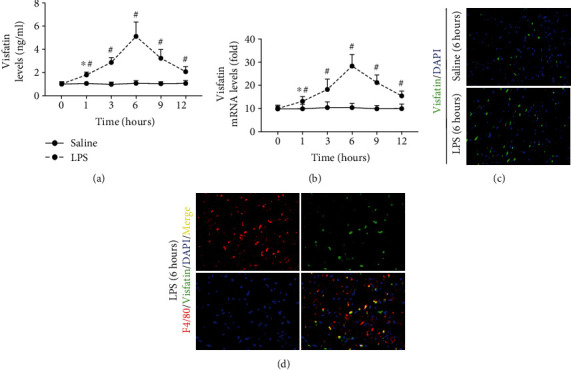
Expression of visfatin in septic mice. (a and b) Dynamic changes in visfatin levels in the serum and heart were detected in mice with LPS-induced septicemia. (c) Cardiac visfatin expression was detected in saline- and LPS-treated mice (200×). (d) Visfatin expression in cardiac macrophages was detected (200×). *N* = 5 in each group. ^∗^*p* < 0.05 vs. the saline group. ^#^*p* < 0.05 vs. the previous group.

The ELISA results showed that compared with the saline group, treatment with LPS increased serum visfatin levels by hour 1; visfatin levels continued to increase at hour 3, peaked by hour 6, and then gradually decreased by hour 9 and hour 12 (Figure 1(a)). Similar trends in cardiac visfatin mRNA levels were observed (Figure 1(b)). The immunofluorescence staining data also showed that treatment with LPS for 6 hours significantly increased visfatin expression in cardiac macrophages (Figures 1(c) and 1(d)).

### 3.2. Visfatin Aggravates LPS-Induced Cardiac Injury in Mice

**Figure 2:**
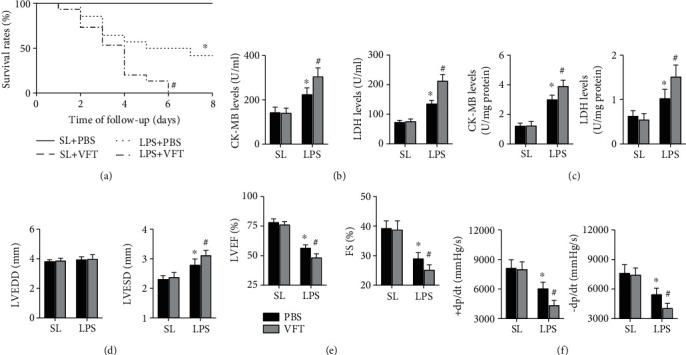
Effects of visfatin on LPS-induced cardiac injury in mice. (a) The survival rates in the four groups were analyzed during follow up; *N* = 10 − 15 in each group. (b and c) The expression levels of CK-MB and LDH in both the serum and heart were examined. (d–f) LVEDD, LVESD, LVEF, FS, +dp/dt max, and −dp/dt max in each group were detected. *N* = 5 − 10 in each group. ^∗^*p* < 0.05 vs. the saline + PBS group. ^#^*p* < 0.05 vs. the LPS + PBS group. SL means saline; VFT means visfatin.

At the 8-day follow up, pretreatment with visfatin significantly increased mortality in mice with LPS-induced septicemia (Figure 2(a)). Visfatin exhibited increased expression levels in both the serum and heart in LPS-treated mice (Figures 2(b) and 2(c)). In addition, septic mice that received visfatin exhibited aggravation of cardiac dysfunction, with further increases in LVESD and further decreases in LVEF, FS, +dp/dt, and −dp/dt, although no effects on LVEDD were observed (Figures 2(d)–2(f)).

### 3.3. Visfatin Amplifies Cardiac Inflammation in LPS-Treated Mice

**Figure 3:**
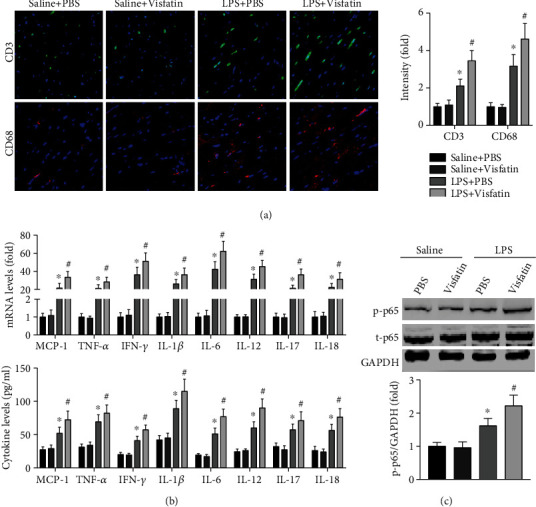
Effects of visfatin on cardiac inflammation in LPS-treated mice. (a) Cardiac CD3+ cells and CD68+ cells in the four groups were examined. (b) The expression of MCP-1, TNF-*α*, IFN-*γ*, IL-1*β*, IL-6, IL-12, IL-17, and IL-18 in the heart and serum was detected. (c) The activation of the NF-*κ*B p65 pathway was measured. *N* = 5 − 6 in each group. ^∗^*p* < 0.05 vs. the saline + PBS group. ^#^*p* < 0.05 vs. the LPS + PBS group.

Immunofluorescence staining showed that the infiltration of both CD3+ cells and CD68+ cells into the heart was significantly increased (Figure 3(a)). In addition, the mRNA and protein expression levels of inflammation-related factors, including MCP-1, TNF-*α*, IFN-*γ*, IL-1*β*, IL-6, IL-12, IL-17, and IL-18, in the heart and serum were significantly increased by visfatin treatment in LPS-treated mice (Figure 3(b)). Visfatin also further elevated NF-*κ*B p65 pathway phosphorylation induced by LPS (Figure 3(c)).

### 3.4. Treatment with Visfatin Increases LPS-Induced Myocardial Cell Apoptosis in Mice

**Figure 4:**
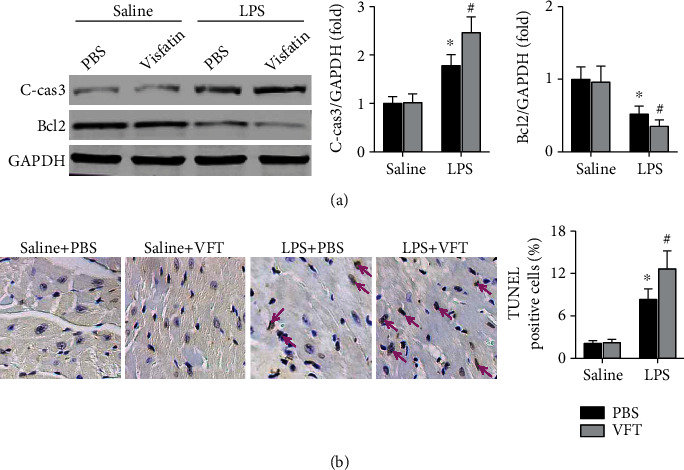
Effects of visfatin on LPS-induced myocardial cell apoptosis in mice. (a) The expression of cleaved caspase3 and Bcl2, apoptosis-related proteins, was detected. (b) TUNEL-positive cells were examined. *N* = 5−6 in each group. ^∗^*p* < 0.05 vs. the saline + PBS group. ^#^*p* < 0.05 vs. the LPS + PBS group. VFT means visfatin.

Cardiac expression of cleaved caspase3, a proapoptotic protein, was increased in visfatin-pretreated septic mice compared with septic mice, while the expression of the proapoptotic protein Bcl2 was decreased (Figure 4(a)). Visfatin further increased the percentage of TUNEL-positive cells in LPS-treated mice, which indicated apoptotic myocardial cells (Figure 4(b)).

### 3.5. Inhibition of the NF-*κ*B p65 Pathway Improves Cardiac Dysfunction in Visfatin-Treated Septic Mice

**Figure 5:**
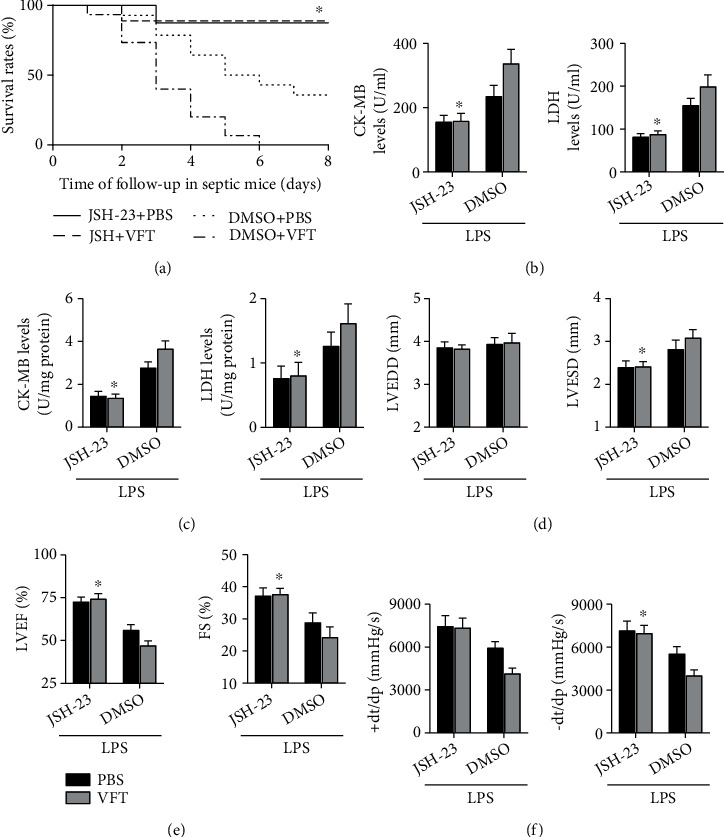
Effects of JSH-23 on LPS-induced cardiac injury and dysfunction in mice. (a) The effects of JSH-23 on the survival rates of visfatin-treated septic mice were measured, *N* = 10 − 15 in each group. (b and c) Cardiac injury markers in the serum and heart were measured. (d–f) Cardiac structure and function in each group were measured. *N* = 5 − 10 in each group. ^∗^*p* < 0.05 vs. the LPS + DMSO + VFT group. VFT means visfatin.

The mortality rate of visfatin-treated septic mice was significantly reduced by JSH-23, an inhibitor of the NF-*κ*B p65 pathway (Figure 5(a)). The visfatin-mediated increase in cardiac injury markers, including CK-MB and LDH, in both the heart and serum was reversed by JSH-23 (Figures 5(b) and 5(c)). The effects of visfatin on the aggravation of LPS-induced cardiac dysfunction were also significantly improved after administration of JSH-23 (Figures 5(d)–5(f)).

### 3.6. JSH-23 Alleviates the Cardiac Inflammatory Response and Myocardial Cell Apoptosis

**Figure 6:**
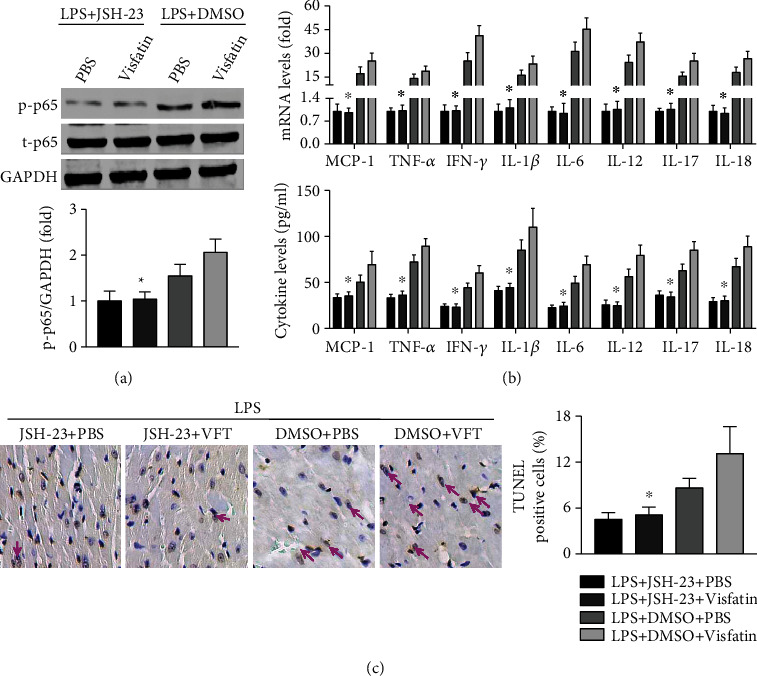
Effects of JSH-23 on cardiac inflammation and myocardial cell apoptosis. (a) Phosphorylation of NF-*κ*B p65 pathway components was detected. (b) Inflammatory mediators in the heart and serum were examined. (c). The numbers of TUNEL-positive myocardial cells were quantified. *N* = 5 − 10 in each group. ^∗^*p* < 0.05 vs. the LPS + DMSO + VFT group.

Treatment with JSH-23 decreased the activation of the NF-*κ*B p65 pathway in visfatin-treated septic mice (Figure 6(a)). The expression levels of MCP-1, TNF-*α*, IFN-*γ*, IL-1*β*, IL-6, IL-12, IL-17, and IL-18 were all decreased in both the heart and serum in septic mice treated with JSH-23 compared with those not treated with JSH-23 (Figure 6(b)). Fewer apoptotic cells were observed when JSH-23 was administered to visfatin-treated septic mice (Figure 6(c)).

## 4. Discussion

As an important adipokine, visfatin has been demonstrated to be involved in the processes of a variety of cardiovascular diseases, while its role in cardiac injury in sepsis has not been reported. In this study, we found that visfatin expression was elevated in septic mice, and that visfatin may be secreted by macrophages. Treatment with visfatin exacerbates cardiac inflammation and aggravates cardiac injury and cardiac dysfunction. These effects are reversed when the NF-*κ*B p65 pathway is inhibited by JSH-23. Our study found that visfatin may amplify cardiac inflammation through the NF-*κ*B p65 pathway and aggravate LPS-induced cardiac injury. Visfatin may be a potential target for the prevention of cardiac injury in sepsis in the clinic.

LPS is an important pathogenic factor in sepsis that can cause damage to a variety of tissues and organs, and its process can be regulated by visfatin. Yao et al. reported that immune cell infiltration and splenic cell apoptosis were significantly increased after LPS administration but were further aggravated after visfapin treatment [17]. Xiao et al. found that visfatin further aggravates LPS-mediated periodontal injury, exacerbates periodontitis symptoms and affects prognosis [18]. In addition, treatment with visfatin can aggravate LPS-induced intestinal injury in both rats and mice [19, 20]. These studies suggest that visfatin may aggravate LPS-induced tissue damage. In another study, Luo et al. reported that visfatin treatment unexpectedly alleviated LPS-induced acute lung injury [15]. The reason for the inconsistent results may be related to different organs. In this study, we examined the regulatory effect of visfatin on septic cardiac injury and found that visfatin further increased the mortality of mice, upregulated the expression of markers of myocardial injury in both the serum and heart, and worsened cardiac dysfunction. These results suggest that visfatin exacerbates LPS-induced cardiac injury.

Various pathological effects, such as the inflammatory response, oxidative stress, calcium overload, and ferroptosis, are involved in the development of LPS-induced tissue damage [1, 21]. Due to the poor tolerance of cardiomyocytes to the inflammatory response, an enhanced inflammatory response can activate programmed death mechanisms in cardiomyocytes, including caspase-dependent and caspase-independent pathways, leading to cardiomyocyte death and cardiac dysfunction [1]. Therefore, inflammation plays a crucial role in sepsis-induced cardiac injury. Visfatin can lead to insulin resistance, which results in the progression of diabetes, affecting lipid metabolism, which is associated with the development of obesity and the invasion of immune cells into tissues, leading to an increased inflammatory response [15, 20, 22]. It has been widely reported that visfatin participates in sepsis-induced tissue injury by regulating the inflammatory response [1, 20–22]. Previous studies have found that the activation of hepatic stellate cells and the regulation of breast cancer by visfatin are mediated by macrophages [23, 24]. Reduction of visfatin expression can significantly reduce T lymphocyte activation, thereby improving spinal cord injury and reducing the disability rate [25]. In rheumatic diseases, visfatin can aggravate Th1/Th2 imbalance, thereby promoting disease progression and joint injury [26]. These studies suggest that both macrophages and T lymphocytes are downstream signals of visfatin and mediate the regulatory effect of visfatin on inflammatory responses. Therefore, to explain the mechanism by which visfatin regulates cardiac injury in LPS-treated mice, the regulation of inflammation by visfatin was measured. The infiltration of inflammatory cells into the heart was first detected, and the results showed that the expression of both CD3+ T lymphocytes and F4/80+ macrophages was significantly increased. The levels of inflammatory mediators were further examined, and we found that treatment with visfatin further increased the expression of a variety of proinflammatory factors. These results suggest that visfatin is involved in the regulation of sepsis-induced cardiac injury by regulating the inflammatory response.

NF-*κ*B p65 is an important intracellular nuclear transcription factor, and phosphorylation is its activation mode, mainly involved in the immune response, stress response and regulation of apoptosis and other biological effects. An increasing number of studies have confirmed that NF-*κ*B p65 can regulate the infiltration and differentiation of various immune cells, including macrophages and lymphocytes, into tissues and magnify the inflammatory response to participate in the regulation of tissue injury.

An increasing number of studies have confirmed that visfatin is closely related to the NF-*κ*B p65 signaling pathway. In a previous study, Wang et al. found that activation of the NF-*κ*B p65 signaling pathway releases IL-6 and promotes metastasis of osteosarcoma cells [27]. Kanda et al. reported that the phosphorylation of NF-*κ*B p65 signaling may mediate the release of CXC chemokine ligand 8 (CXCL8), CXCL10, and CXCL20 in the presence of visfatin, which is associated with cuticle formation [28]. Decreased visfatin expression downregulated the activation of the NF-*κ*B p65 pathway, and cell invasiveness was significantly reduced [29]. In addition, administration of visfatin also further activated the NF-*κ*B p65 signaling pathway in LPS-treated mice and cells [30, 31]. These studies suggest that the NF-*κ*B p65 pathway may be an important downstream signal of visfatin and that the NF-*κ*B p65 pathway may regulate LPS-induced cardiac injury and cardiac dysfunction.

To confirm the above speculation, the small molecule compound JSH-23, a specific inhibitor of the NF-*κ*B p65 signaling pathway that was reported in a previous study, was administered to visfatin-treated septic mice. The results showed that treatment with JSH-23 significantly reduced mortality rates in septic mice pretreated with visfatin at the 8-day follow up. In addition, the expression levels of cardiac injury markers and cardiac dysfunction were also reversed in mice treated with JSH-23. These results suggest that JSH-23 reverses the regulatory effect of visfatin on cardiac injury and cardiac dysfunction induced by LPS. At the same time, the phosphorylation level of NF-*κ*B p65 was decreased; the levels of inflammatory markers in both the heart and serum were reduced significantly, and myocardial cell apoptosis was alleviated, suggesting that JSH-23 improved the cardiac inflammatory response and myocardial apoptosis. These results suggest that the regulatory effect of visfatin on sepsis-induced cardiac injury and cardiac dysfunction is mediated by the NF-*κ*B p65 pathway. NF-*κ*B p65 pathway has been reported to play an important role in the regulation of inflammatory response, and its activation can promote the release of a variety of proinflammatory factors. Therefore, the expression of a variety of cardiac injury related proinflammatory factors, including MCP-1, TNF-*α*, IFN-*γ*, IL-1*β*, IL-6, IL-12, IL-17, and IL-18, were detected; the results showed that treatment with visfatin furthere increased these cytokines expression in LPS-induced septic mice, and these effects were abolished by JSH-23. These results suggest that visfatin may be involved in the regulation of inflammatory response through the NF-*κ*B p65 pathway.

In summary, we found that visfatin may further activate the NF-*κ*B p65 pathway to amplify the inflammatory response, promote myocardial cell apoptosis, and aggravate cardiac injury and cardiac dysfunction. Visfatin may be a potential target for interventions for sepsis-induced cardiac injury.

## Figures and Tables

**Table 1:** Primers used in this study.

Genes	Forward primer (5′ →3′)	Reverse primer (5′ →3′)
Visfatin	AATGTCTCCTTCGGTTCTGG	CCGCTGGTGTCCTATGTAAA
MCP-1	CTTCTGTGCCTGCTGCTCAT	CGGAGTTTGGGTTTGCTTGTC
TNF-*α*	CCCAGGGACCTCTCTCTAATC	ATGGGCTACAGGCTTGTCACT
IFN-*γ*	ACTGGCAAAAGGATGGTGAC	TGAGCTCATTGAATGCTTGG
IL-1*β*	GGGCCTCAAAGGAAAGAATC	TACCAGTTGGGGAACTCTGC
IL-6	AGTTGCCTTCTTGGGACTGA	TCCACGATTTCCCAGAGAAC
IL-12	AGTTTGGCCAGGGTCATTCC	TCTCTGGCCGTCTTCACCAT
IL-17	TCCAGAAGGCCCTCAGACTA	AGCATCTTCTCGACCCTGAA
IL-18	ATGCTTTCTGGACTCCTGCC	GTCTGGTCTGGGGTTCACTG
GAPDH	AACTTTGGCATTGTGGAAGG	CACATTGGGGGTAGGAACAC

## Data Availability

Our data is available to scientific researchers except for commercial purposes.
